# Circular RNA circANAPC2 mediates the impairment of endochondral ossification by miR‐874‐3p/SMAD3 signalling pathway in idiopathic short stature

**DOI:** 10.1111/jcmm.16419

**Published:** 2021-03-13

**Authors:** Xijuan Liu, Zhi Du, Xuan Yi, Tianle Sheng, Jinghong Yuan, Jingyu Jia

**Affiliations:** ^1^ Department of Pediatrics The Second Affiliated Hospital of Nanchang University Nanchang City China; ^2^ Department of Orthopaedics The Second Affiliated Hospital of Nanchang University Nanchang City China; ^3^ Department of Molecular laboratory The Second Affiliated Hospital of Nanchang University Nanchang City China

**Keywords:** circular RNA, endochondral ossification, idiopathic short stature

## Abstract

Idiopathic short stature (ISS) is a main reason for low height among children. Its exact aetiology remains unclear. Recent findings have suggested that the aberrant expression of circRNAs in peripheral blood samples is associated with many diseases. However, to date, the role of aberrant circRNA expression in mediating ISS pathogenesis remains largely unknown. The up‐regulated circANAPC2 was identified by circRNA microarray analysis and RT‐qPCR. Overexpression of circANAPC2 inhibited the proliferation of human chondrocytes, and cell cycle was arrested in G1 phase. The expressions of collagen type X, RUNX2, OCN and OPN were significantly down‐regulated following circANAPC2 overexpression. Moreover, Von Kossa staining intensity and alkaline phosphatase activity were also decreased. Luciferase reporter assay results showed that circANAPC2 could be targeted by miR‐874‐3p. CircANAPC2 overexpression in human chondrocytes inhibits the expression of miR‐874‐3p. The co‐localization of circANAPC2 and miR‐874‐3p was confirmed in both human chondrocytes and murine femoral growth plates via in situ hybridization. The rescue experiment demonstrated that the high expression of miR‐874‐3p overexpression antagonized the suppression of endochondral ossification, hypertrophy and chondrocyte growth caused by circANAPC2 overexpression. A high‐throughput screening of mRNA expression and RT‐qPCR verified SMAD3 demonstrated the highest different expressions following overcircANAPC2. Luciferase reporter assay results indicated that miR‐874‐3p could be targeted by Smad3, thus down‐regulating the expression of Smad3. Subsequent rescue experiments of SMAD3 further confirmed that circANAPC2 suppresses endochondral ossification, hypertrophy and chondrocyte growth through miR‐874‐3p/Smad3 axis. The present study provides evidence that circANAPC2 can serve as a promising target for ISS treatment.

## 1 INTRODUCTION

Idiopathic short stature (ISS) is diagnosed by a height of less than two standard deviation scores in children after the exclusion of identifiable diseases, including systemic diseases, small for gestational infant, psychological disorders, overt hormone deficiency, nutritional imbalance and chromosomal abnormalities.^1^ The patients with ISS often present some problems in behaviour, self‐perception, social adjustment, school competence and attention.^2^, ^3^ Although the pathogenesis of ISS has been studied over past decades, its underlying cause may be complicated and remains largely unclarified.^1^ At present, recombinant human growth hormone (rhGH) has been used to treat ISS patients owing to the unknown aetiology. Silvers et al^4^ reported there are beyond 500 000 ISS children who need to be treated by rhGH and the expense on ISS treatment would exceed $10 billion per year in the United States. Unlike growth hormone deficiency‐related short stature, ISS patients do not demonstrate the absence of growth hormones. Therefore, the treatment effect of recombinant human growth hormones on ISS varies considerably between studies.^5^, ^6^, ^7^, ^8^ Moreover, high‐dose rhGH treatment seems to be related to some side effects, such as bone and cartilage cancer, cerebrovascular accidents, hyperglycaemia and hyperinsulinaemia.^8^, ^9^, ^10^

Ying et al^8^ found that the growth velocity of ISS patients (9.55 ± 0.13 cm) in the first year clearly increased after rhGH treatment compared to that before treatment. Kim et al^11^ also observed that rhGH treatment for prepubertal ISS patients presented a significant improvement in annual growth rate of height in a phase III randomized trial. However, Van et al^6^ observed that although the height SDS in ISS patients was significantly improved after rhGH treatment, they showed an accelerated bone maturation. As a result, no significant difference in adult height was identified between the rhGH treatment and the untreated control group (169.7 ± 4.2 and 168.8 ± 3.8, respectively). Given the efficacy of rhGH treatment on the GH‐IGF‐I axis, researchers must pay more attention to the issue of rhGH safety. Carel et al^9^ assessed the long‐term mortality of 6928 children, including 5162 patients with idiopathic isolated GH deficiency, 534 patients with neurosecretory dysfunction and 871 patients with ISS and 335 patients with small for gestational age after rhGH treatment. They observed an increased risk of mortality for bone and cartilage cancer, and cerebrovascular accidents after rhGH treatment. Ying et al^8^ reported the side effects of rhGH treatment on ISS patients, such as 21.5% chance of hyperglycaemia and 17% chance of hyperinsulinaemia. These studies suggest that the pathogenesis of ISS needs to be urgently elucidated to improve methods for treating the condition.

Circular RNAs (circRNAs) are a group of non‐coding RNAs that are highly conserved and commonly found in mammalian cells.^12^, ^13^, ^14^ Their stability is high compared to other non‐coding lncRNAs and miRNAs owing to their closed circular structures. CircRNAs could suppress the functions of their downstream gene by acting as miRNA sponges.^15^ Their aberrant expression has been related to many diseases such as cardiovascular diseases, diabetes mellitus, osteoarthritis, cancers, pre‐eclampsia and neurological disorders.^16^, ^17^, ^18^ CircRNAs have demonstrated potential applications as disease biomarkers and novel therapeutic targets.^19^ Li et al^20^ observed that circ_0136474 inhibited cell proliferation and accelerated cell apoptosis via acting as a sponge with miR‐127‐5p in osteoarthritis. Zhou et al^21^ observed that circRNAs.33186, as miRNA sponges, significantly increased MMP‐13 expression by directly binding to and inhibiting miR‐127‐5p in osteoarthritis. Their findings demonstrate circRNAs.33186 can be considered as a potential drug target in osteoarthritis therapy. Although circRNAs have demonstrated potential applications for uncovering the molecular mechanisms of disease, it remains unknown whether circRNAs can mediate the pathogenesis of ISS.

In this research, we assessed the different expression patterns of circRNAs in ISS patients and normal controls. We found that circANAPC2 (hsa_circRNAs_0003129) can suppress endochondral ossification, hypertrophy and chondrocyte proliferation via a novel circANAPC2/miR‐874‐3p/SMAD3 regulatory pathway. This study reveals that circANAPC2 can serve as a promising target for ISS treatment.

## 2 MATERIALS AND METHODS

### 2.1 Study patient characteristics

Sixty‐eight pairs of ISS patients and age‐/gender‐matched healthy individuals were collected at the Second Affiliated Hospital of Nanchang University (China). The ISS patients comprised 32 males and 36 females ranging from 4 to 12 years with an average age of 8.92 ± 0.34 years and with height ranging from 88.71 to 142.2 cm (mean height of 120.2 ± 1.72 cm). The matched control individuals comprised 35 females and 33 males, with a mean age of 8.39 ± 0.28 years (ranging from 4 to 12 years) and a mean height of 132.1 ± 1.57 cm (ranging from 103.9 to 150.9 cm, Table 1). Patients exhibiting the following conditions were excluded: (a) abnormal hormone levels (a growth hormone peak of less than 6 ng/mL) or disturbance of thyroid function and puberty; (b) small for gestational age (size and weight at birth were below the 10th percentile); (c) chronic exposure to environmental contaminants and health conditions that can affect human growth; (d) cytogenetically detected chromosomal aberrations; (e) skeletal anomalies or dysmorphic features; and (f) whole‐exome sequencing. Sixty‐eight pairs of blood specimens were withdrawn from the study patients from October 2016 to March 2019, snap‐frozen in liquid nitrogen and then kept at −80°C for long‐term preservation. Four specimen pairs were used for circRNA microarray analysis, while all 136 specimens were used to validate circRNAs expression by quantitative real‐time polymerase chain reaction (qRT‐PCR). Ethical approval was obtained from the Ethics Committee of the Second Affiliated Hospital of Nanchang University. The parent and/or guardian signed a written consent form prior to the child's participation.

**TABLE 1:** The information of ISS patients and normal control individuals (NC)

Category	Males	Females	Age /y (mean ± SD)	Age range/y	Height /cm (mean ± SD)	Height range/cm
ISS	32	36	8.92 ± 0.34	4‐12	120.2 ± 1.72	88.71 −142.2
NC	33	35	8.39 ± 0.28	4‐12	132.1 ± 1.57	103.9 −150.9

Note

Age, ISS vs NC (*P* =.234); and height, ISS vs NC (*P* =.000).

### 2.2 RNA preparation and microarray hybridization

RNA samples (n = 4 per group) were prepared with Eastep® Super RNA Extraction Kit (Promega (Beijing) Biotech Co., Ltd.), followed by RNase R (Epicentre) digestion to eliminate linear RNAs and enrich circRNAs in the RNA samples. The purity and yields of RNA samples were evaluated using a NanoDrop spectrophotometer (ND‐1000; NanoDrop, Inc). Then, the enriched circRNAs were amplified and transcribed into fluorescence‐labelled coding RNAs using a random priming approach (Super RNA Labeling Kit; Arraystar). The labelled coding RNAs were then hybridized onto the Human circRNAs Array v2 (8 × 15 K, Arraystar). After washing, a G2505C Microarray Scanner System (Agilent Technologies, Inc) was used to perform the microarray analysis.

### 2.3 Differential expression analysis of circRNAs

Microarray data processing was carried out using the Agilent's Feature Extraction version 11.0.1.1 software. The expression levels of circRNAs were measured by quantile normalization method using the Quantile algorithm and limma package version 3.11 (https://www.bioconductor.org/packages/release/bioc/html/limma.html) in R software version 3.6.0. The expression profiles of circRNAs were then classified into control and ISS groups. The differently expressed circRNAs between the control and ISS groups were determined through volcano plot filtering. Statistical significance level was set at a fold‐change value of greater than 2.0 and a false discovery rate (FDR)–corrected P‐value of less than 0.05. Hierarchical cluster analysis of the differential expression patterns of circRNAs was carried out using the R program.

### 2.4 In silico and bioinformatics analyses

Given that circRNAs can alter gene expression levels by acting as miRNA sponges, Starbase (http://starbase.sysu. edu.cn/) and Circinteractome database (http://circinteractome.nia.nih.gov/) were employed to predict the target genes of miR‐874‐3p and circANAPC2 (hsa_circRNAs_0003129), respectively. Cytoscape (http://www. cytoscape.org/) was applied to build circRNAs‐miRNA‐mRNA interaction network. KEGG pathway analyses were carried out on these genes through DAVID version 6.8 (https://david. ncifcrf.gov/). A *p*‐value of < 0.05 and the enrich factor were adopted to identify and rank the corresponding signalling pathways.

Gene ontology (GO) enrichment analysis (http://www.geneongoloty.org/) was used to assess the biological processes (BP), molecular functions (MF) and cellular components (CC) of the identified circRNA targets according to their target mRNAs. Meanwhile, KEGG analysis was carried out using DAVID in order to identify the molecular pathways. The enrichment scores of ‐log10 (*P*‐value) was used as cut‐off value in both GO and KEGG analysis.

### 2.5 Validation of differentially expressed circRNAs by qRT‐PCR

Total RNAs were isolated from the frozen tissues using Eastep® Super RNA Extraction Kit (Promega (Beijing) Biotech Co., Ltd.) and then reversed‐transcribed using PrimeScript™ RT reagent Kit with gDNA Eraser (TaKaRa). QRT‐PCR was conducted on an Applied Biosystems QuantStudio 6 RT‐PCR system (Thermo Fisher Scientific, Inc) using the TB Green® Premix Ex Taq™ II (TaKaRa). The primers used are presented in Table 2. The qRT‐PCR conditions for circANAPC2 amplification were as follows: 10 minutes at 95°C, followed by 40 cycles of 10 seconds at 95°C and 34 seconds at 60°C. Glyceraldehyde 3‐phosphate dehydrogenase (GAPDH) was employed as the internal control. Each assay was performed in triplicate. The 2^‐ΔΔCt^ method was used to determine the relative mRNA expression levels of target genes.

**TABLE 2:** Primers used for qRT‐PCR analysis of circRNA, miRNA and mRNA levels

Target ID	Primer sequence 5’‐3’
circANAPC2	F: GGAGGTCCCCGAGGATATCAG
	R: GGACAGGATGACAGCGTAGACC
CircAXIN1	F: GTGCCCCTACCTCACATTCC
	R: CTCACCTTCCTCCTCCATGC
CircUBE2J2	F: GAAAGACCCGGTGCCTTACA
	R: CTTGCCAATGCCATGACTGG
Actin	F: AGCACAGAGCCTCGCCTTTG
	R: CTTCTGACCCATGCCCACCA
miR‐874‐3p	F: GAACTCCACTGTAGCAGAGATGGT
	R: CATTTTTTCCACTCCTCTTCTCTC
miR‐647	F: GTGTTGGCCTGTGGCTG
	R: CTGACCCTCCCTCCTGC
U6	F: CTCGCTTCGGCAGCACA
	R: AACGCTTCACGAATTTGCGT
SMAD3	F: AGTGGAGCTGACACGGAGAC
	R: AGCGAACTCCTGGTTGTTGA
SMAD2	F: ATGTCGTCCATCTTGCCATT
	R: TTTTCTTCCTGCCCATTCTG
IHH	F: GACTCATTGCCTCCCAGAACTG
	R: CCAGGTAGTAGGGTCACATTGC
RUNX2	F: ACTTCCTGTGCTCCGTGCTG
	R: TCGTTGAACCTGGCTACTTGG
COL10	F: GCAGCATTACGACCCAAGAT
	R: CATGATTGAACTCCCTGAAG
OPN	F: CCAGCCAAGGACCAACTACA
	R: AGTGTTTGCTGTAATGCGCC
OCN	F: GCACCACCGTTTAGGGCAT
	R: CGTTCCTCATCTGGACTTTATTTTG
HDAC4	F: CGGTCCAGGCTAAAGCAGAA
	R: TCTGTGACATCCAACGGACG
GAPDH	F: GGAGCGAGATCCCTCCAAAAT
	R: GGCTGTTGTCATACTTCTCATGG

### 2.6 Cell culture

We purchased the human chondrocytes from the Procell life science technology Co., Ltd. and then cultured in DMEM (Gibco, Thermo Fisher Scientific, Inc) containing 10% FBS (Gibco) before being maintained at 37°C in a humidified incubator with 5% CO_2_.

### 2.7 Overexpression vectors circANAPC2 and miR‐874‐3p, and siRNA of circANAPC2

The circANAPC2 plasmid (pHBLV‐CMV‐Cicr‐MCS‐EF1‐zsgreen‐t2a‐puro) and SMAD3 plasmid (pSI‐Check2) were successfully constructed by Hanbio Biotechnology Co., Ltd. (Figure S1). miR‐874‐3p mimics from Guangzhou RiboBio Co., Ltd. were obtained. Human chondrocytes were grown on a 6‐well plate until achieving ~ 80% confluence. The circANAPC2 plasmid was transfected to a 6‐well plate using Lipofectamine 3000 (Invitrogen). The empty vector was employed as the negative control (NC). The transfection efficiency of circANAPC2 was evaluated by using a fluorescence microscope (Figure S2). The final concentration of circANAPC2 and SMAD3 plasmid was 50 nmol/L. CircANAPC2 siRNA (ATGAGCAGCTCAAGGACAT) and NC siRNA (AAGTCGGGTCAAGAGAAGC) were supplied by Guangzhou RiboBio Co., Ltd. The miR‐874‐3p mimic (sense CUGCCCUGGCCCGAGGGACCGA, and antisense UCGGUCCCUCGGGCCAGG GCAG) and NC mimics (sense UUUGUACUACACAAAA GUACUG, and antisense CAGUACUUUU GUGUAGUACAAA) were supplied by Guangzhou RiboBio Co., Ltd. (China). MiR‐874‐3p (5 µL)/NC mimics were transfected to a 6‐well plate using Lipofectamine 2000 (Invitrogen) in compliance with the manufacturer's protocol. The final concentration of miR‐874‐3p/NC mimics was 50 nmol/L. The further experimentation was conducted 72 hours after the cells were transfected. All assays were conducted in triplicate.

### 2.8 Cell proliferation assay

Chondrocytes of overcircANAPC2 were seeded (50%‐60%) in a 6‐well plate and cultured until they reached 70%‐80% confluency. Cell Counting Kit 8 (CCK‐8; TransGen Biotech Co., Ltd) was used to detect chondrocytes proliferation rate in compliance with the manufacturer's protocol. The detailed steps of CCK8 assay were referring to our previous report.^22^

### 2.9 mRNA high‐throughput sequencing

We employed the TruSeq Stranded mRNA LT Sample Prep Kit (Illumina) and constructed the libraries according to the manufacturer's instructions. Transcriptomic sequencing and analysis were carried out at the OE Biotech Co., Ltd. The raw sequences were produced using an Illumina HiSeq X Ten sequencer. High‐quality sequences were filtered by Trimmomatic after removing the low‐quality reads with adapter and ploy‐N. We used the HISAT2 to compare the high‐quality reads and human genome. Cufflink quantitatively evaluated the FPKM value of each gene. HTSeq‐count calculated the read counts of each gene. Differential expression analysis was conducted using the DESeq (2012) R package. A *P*‐value less than .05 and a fold change of greater than 2 were considered as the cut‐off value for GO and KEGG analysis.

### 2.10 Luciferase reporter assay

The circANAPC2 plasmid or its mutation fragments (Figure S3) was cotransfected with miR‐874‐3p mimic into 293T cells using Lipofiter (Hanbio Biotechnology) by following the manufacturer's instructions. The graphical presentation vector structure was preferred. Similarly, SMAD3 plasmid (pSI‐Check2, Hanbio Biotechnology) or its mutated fragments (Figure S3) was cotransfected with miR‐874‐3p into 293T cells. After transfection for 48 hours, dual‐luciferase reporter assays (Promega Corp.) were conducted to measure the Renilla and firefly luciferase activities of the transfected cells.

### 2.11 Western blotting

Total protein was isolated using RIPA lysis buffer (Applygen Technologies Inc). BCA assay (Thermo Fisher Scientific, Inc) was used to determine the protein levels. The protein lysate was then separated through SDS‐PAGE (Beijing Biosynthesis Biotechnology) and transferred onto PDVF membrane. After blocking with skim milk (Solarbio Inc) for 1 hours at room temperature, the membrane was added with anti‐Smad3 (1:2000 dilution; Abcam), anti‐RUNX2 (1:2000 dilution; Abcam), anti‐collagen type X (1:2000 dilution; Abcam), anti‐Indian Hedgehog (1:2000 dilution; Abcam), anti‐OPN (1:2000 dilution; Abcam) and anti‐OCN (1:2000 dilution; Abcam) primary antibodies. After overnight incubation at 4°C, the membrane was rinsed in 1 × TBST (Solarbio Inc) and then incubated with HRP‐labelled rabbit antimouse (1:3000 dilution; Abcam, USA) secondary antibody at room temperature for 1 hours. We employed GAPDH as the internal control (1:3000, Abcam). Additionally, when > 1 protein was presented, the Western blotting membranes were stripped and reprobed with WB Stripping buffer (No: 21059; Thermo Fisher Scientific, USA) and different primary antibodies, respectively. The grey values were measured by Image Lab (v 5.2.1).

### 2.12 In situ hybridization

After incubation with 500 ng/mL of FAM‐labelled probe and Cy3‐labelled probe (has‐circ‐0003129 sequence: 5’‐Cy3‐CCGCCATGTCCTTGAGCTGCTCATACTTCTTGCAGTAAGCCTCCA‐3’, length 45 bp; hsa‐miR‐874‐3p sequence: 5’‐TCGGTCCCTCGGGCCAGG GCAG‐3’, length 21 bp) at 65°C for 48 h, the expression levels of hsa_circRNAs _0003129 and miR‐874‐3p expression (miRCURY LNA miRNA ISH kit, Thermo Fisher, Shanghai, China) in human chondrocytes were examined.

Eight 6‐week‐old C57 mice of either gender weighing 16.4‐22.5 g and eight 10‐week‐old SD rat of either gender weighing 222.7‐300.9 g involved in the study. The mixed feeds (Beijing Keao Xieli Feed Co. Ltd., China) were used to feed the mice in a cage at 23 ± 1°C under a 12:12‐hours light/dark cycle. Femur samples were extracted after the mice were killed 160 mg/kg of pentobarbital via intraperitoneal injection and then kept in a 4% formaldehyde solution. After that, we used a 10% EDTA solution to decalcify the femur samples for 4 weeks. Paraffin was employed embedding the femur samples. The paraffin‐embedded specimens were then cut into 4‐μm thickness sections. Finally, in situ hybridization was performed (miRCURY LNA miRNA ISH kit; Thermo Fisher Scientific, Inc). The ethical approval for animal experimentation was obtained from the Animal Ethics Committee of Nanchang University (Nanchang, China).

### 2.13 Alkaline phosphatase assay and Von Kossa staining

The detailed steps of Von Kossa staining and alkaline phosphatase assay were referring to our previous report.^22^ First, we washed the chondrocytes three times using PBS. Then, the chondrocytes were fixed using 4% paraformaldehyde for 15 min at room temperature. Afterwards, the fixed chondrocyte cells were exposed to a BCIP/NBT Alkaline Phosphatase Color Development Kit (Solarbio Inc) for 48 h in the dark. Meanwhile, mineralized nodules in chondrocytes were evaluated by using Von Kossa staining Kit (Shanghai Gefan Biotechnology Co., Ltd) in compliance with the manufacturer's instructions. A black or dark brown was identified as positive calcium nodules. The red colour was the background.

### 2.14 Statistical analysis

Statistical differences between groups were compared with the unpaired t test or one‐way analysis of variance (ANOVA) followed by Tukey's test. *P‐*values of < .05 (*), <0.01 (**) and < .001 (***) were deemed as statistically significant. SPSS 20.0 (IBM) and GraphPad Prism 5.0 (GraphPad) were employed to conduct the statistical tests.

## 3 RESULT

### 3.1 Bioinformatics analysis of differentially expressed circRNAs

**FIGURE 1:**
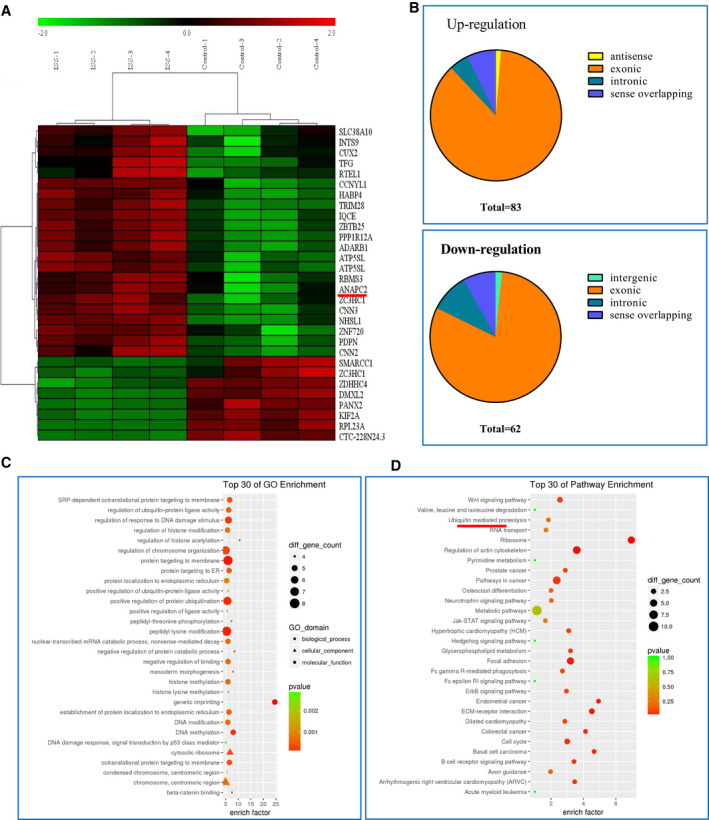
Microarray analysis of differentially expressed circRNAs. A, Four pairs of ISS patients and age‐/sex‐matched controls were screened via circRNA microarray analysis. Thirty differentially expressed circRNAs were shown, including top 22 up‐regulated circRNAs and top 8 down‐regulated circRNAs. B, 145 circRNAs were significantly differentially expressed, including 83 up‐regulation and 62 down‐regulation. C, D, The top 30 KEGG pathway and GO enrichment are exhibited. The ubiquitin‐mediated proteolysis and Wnt signalling pathway are closely associated with chondrocyte proliferation, hypertrophy and endochondral ossification. CircRNAs, circular RNAs; KEGG, Kyoto Encyclopedia of Genes and Genomes; GO, Gene ontology; and Wnt, wingless/integrated

ISS patients and age‐/gender‐matched control individuals (n = 4 per group) were subjected to circRNA microarray analysis (Figure 1A). A total of 145 circRNAs were differentially expressed in ISS patients (*P* <.05, |logFC|‐value > 2.0). Compared to normal individuals, in ISS patients, the expression levels of 83 circRNAs were up‐regulated and those of 62 were down‐regulated (Figure 1B).

The 145 differentially expressed circRNAs were further enriched with GO and KEGG analyses, as they can modulate the expression levels of their target genes. The top 30 KEGG pathway and GO enrichment are shown in Figure 1C and 1D, respectively. Several studies ^23^, ^24^, ^25^, ^26^ have confirmed that the ubiquitin‐mediated proteolysis and Wnt signalling pathway, from the top 30 KEGG pathway, are closely associated with chondrocyte proliferation, hypertrophy and endochondral ossification. The circAXIN1 (hsa_circ_0037160), circGSK3B (hsa_circ_0008797) and circSENP2 (hsa_circRNAs_403044) were involved in Wnt signalling pathway, and circUBE2J2 (hsa_circ_0009246) and circANAPC2 (hsa_circ_0003129) were ubiquitin‐mediated proteolysis. We selected the up‐regulated circRNAs (circAXIN1, circUBE2J2 and circANAPC2) to further verify the outcome of microarrays.

### 3.2 CircANAPC2 was up‐regulated in ISS

**FIGURE 2:**
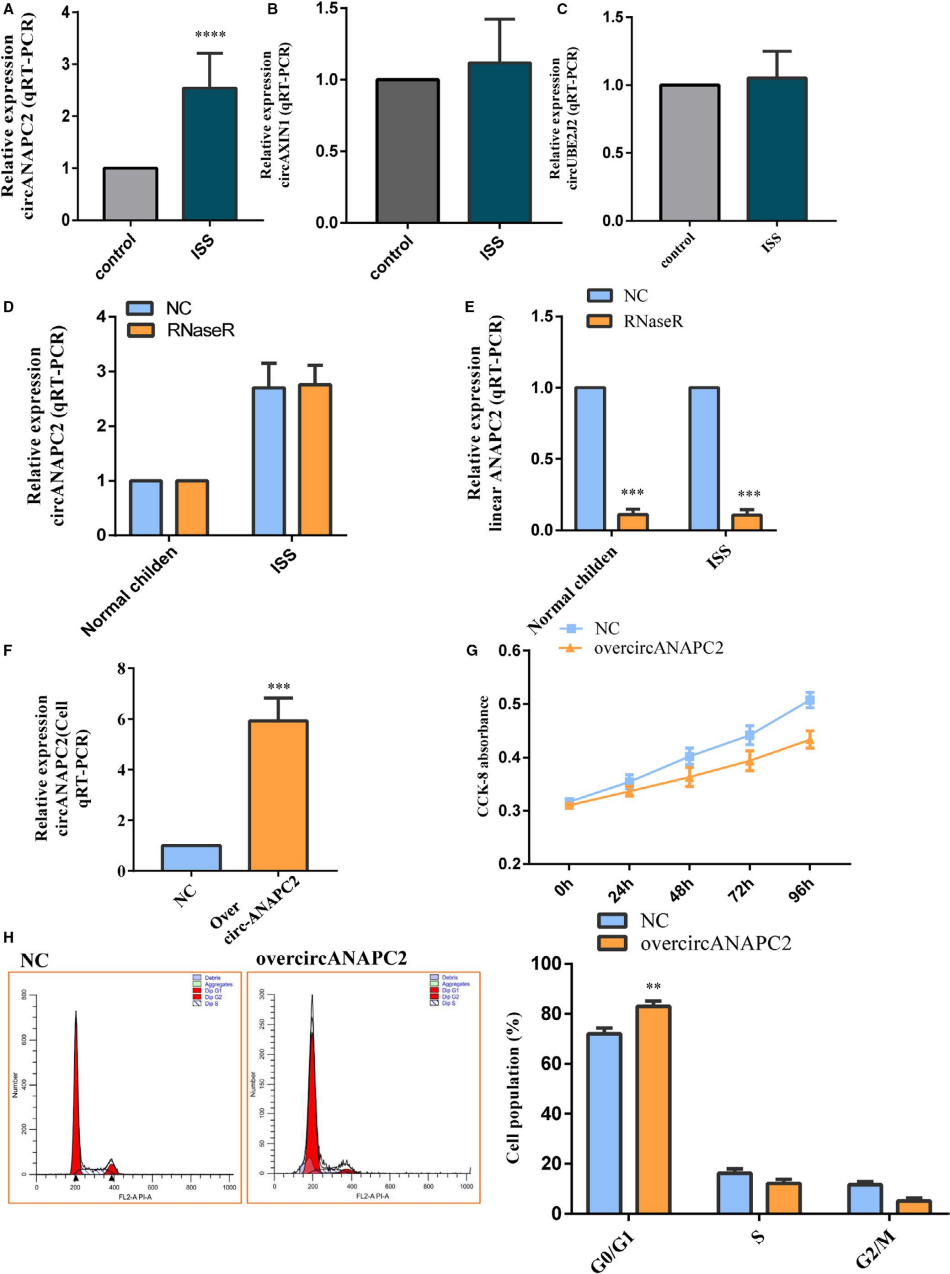
CircANAPC2 suppressed chondrocyte proliferation. A, The up‐regulated circANAPC2 in ISS patients was verified by RT‐qPCR. B,C, No significant difference was shown in the circAXIN1 and circUBE2J2 between the ISS patients and normal control individuals. D, No significant difference in the circANAPC2 was observed between the RNase R and normal control groups. E, The linear ANAPC2 was significantly reduced after the RNase R treatment. Meanwhile, the expression of linear ANAPC2 did not present a significant difference between the ISS sample and the normal control. This indicated that the circANAPC2, but not linear ANAPC2, was up‐regulated in ISS. F, CircANAPC2 was successfully overexpressed via overcircANAPC2 vectors. G, CCK8 revealed that human chondrocyte proliferation was inhibited following overcircANAPC2. H, Flow cytometry assays indicated that the cell cycle was arrested in the G1 phase. The data are presented as the mean ± SD. n = 3. *t* Test, ^**^ *P* <.01, ^***^ *P* <.001 vs control. ISS, idiopathic short stature; ANAPC2, anaphase promoting complex subunit 2; ISS, idiopathic short stature; and CCK‐8, Cell Counting Kit‐8

CircAXIN1, circUBE2J2 and circANAPC2 expressions were assessed by RT‐qPCR in 136 samples, including 68 ISS patients and 68 control individuals. Only the expression of circANAPC2 presented significant up‐regulation in ISS patients compared to control individuals (Figure 2A‐C). Additionally, the host gene (ANAPC2) of circANAPC2 is also an important cell cycle protein and involved in the cell cycle among the top 30 KEGG pathway. Thus, the circANAPC2 was considered as a candidate gene to reveal ISS pathogenesis. Our outcome of RT‐qPCR showed excellent reliability with the circRNA microarray (Figure 2A). No obvious difference was found between the normal control and RNase R groups (Figure 2D). In contrast, the linear ANAPC2 was significantly reduced after the RNase R treatment (Figure 2E). Moreover, the expression levels of linear ANAPC2 were relatively similar between ISS and control groups. This indicated that the circANAPC2, but not linear ANAPC2, was up‐regulated in ISS.

### 3.3 CircANAPC2 suppressed chondrocyte proliferation, hypertrophy and endochondral ossification

CircANAPC2 was overexpressed following transfection with pHBLV‐circANAPC2 in human chondrocytes to analyse the role of circANAPC2 on chondrocyte physiology (Figure 2F). Up‐regulation of circANAPC2 inhibited the proliferation of human chondrocytes as shown by CCK‐8 (Figure 2G). Meanwhile, flow cytometric analysis indicated that pHBLV‐circANAPC2‐transfected chondrocytes had different cell phases. The quantity of G0/G1‐phase cells was noticeably higher in pHBLV‐circANAPC2 group than in NC group (Figure 2H). Besides, fewer cells were arrested at the S and G2/M phases, implying that G1 cell cycle arrest is more obvious in pHBLV‐circANAPC2 group than in NC group.

**FIGURE 3:**
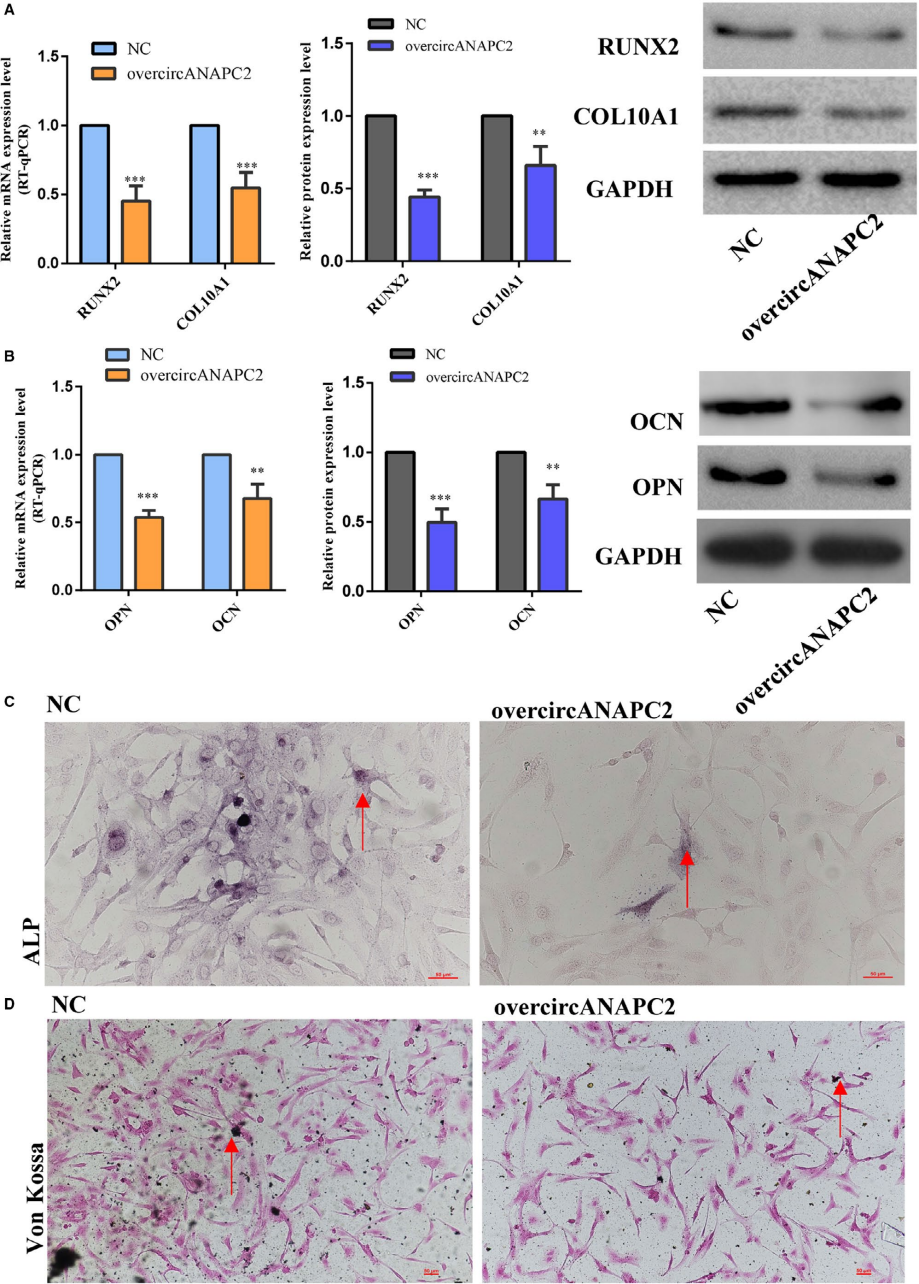
CircANAPC2 suppressed chondrocyte hypertrophy and endochondral ossification. A, The mRNA and protein expressions of collagen type X and RUNX2 were down‐regulated following overcircANAPC2. B, The mRNA and protein expressions of OCN and OPN were also down‐regulated following overcircANAPC2. C, ALP activity was decreased following overcircANAPC2. D, Von Kossa staining demonstrated reduced mineralization following overcircANAPC2. These results indicated that the up‐regulated circANAPC2 can markedly suppress chondrocyte proliferation, hypertrophy and endochondral ossification. The data are presented as the mean ± SD. n = 3. *t* Test, ^**^ *P* <.01, ^***^ *P* <.001 vs control. mRNA, messenger RNA; RUNX2, runt‐related transcription factor 2; ALP, alkaline phosphatase; ANAPC2, anaphase promoting complex subunit 2; OPN, osteopontin; and OCN, osteocalcin

To evaluate the effects of circANAPC2 on chondrocyte hypertrophy and endochondral ossification, the expressions of collagen type X, RUNX2, OCN, OPN, the staining of Von Kossa and alkaline phosphatase (ALP) were evaluated after circANAPC2 overexpression. The outcome of a Western blot analysis and RT‐qPCR confirmed that the expression levels of RUNX2 and collagen type X were significantly down‐regulated following circANAPC2 overexpression. This suggests that chondrocyte hypertrophy was suppressed (Figure 3A **)**. As shown in Figure 3B, the expressions of the osteogenic genes (OCN and OPN) were down‐regulated by RT‐qPCR and Western blot analysis after circANAPC2 overexpression. Meanwhile, ALP activity was also decreased (Figure 3C **)**. Additionally, Von Kossa staining demonstrated reduced mineralization (Figure 3D **)**. These results indicated that overexpression of circANAPC2 can markedly suppress endochondral ossification, hypertrophy and chondrocyte proliferation (Figure 3C and 3D **)**.

### 3.4 CircANAPC2 suppressed endochondral ossification, hypertrophy and chondrocyte proliferation by regulating miR‐874‐3p

**FIGURE 4:**
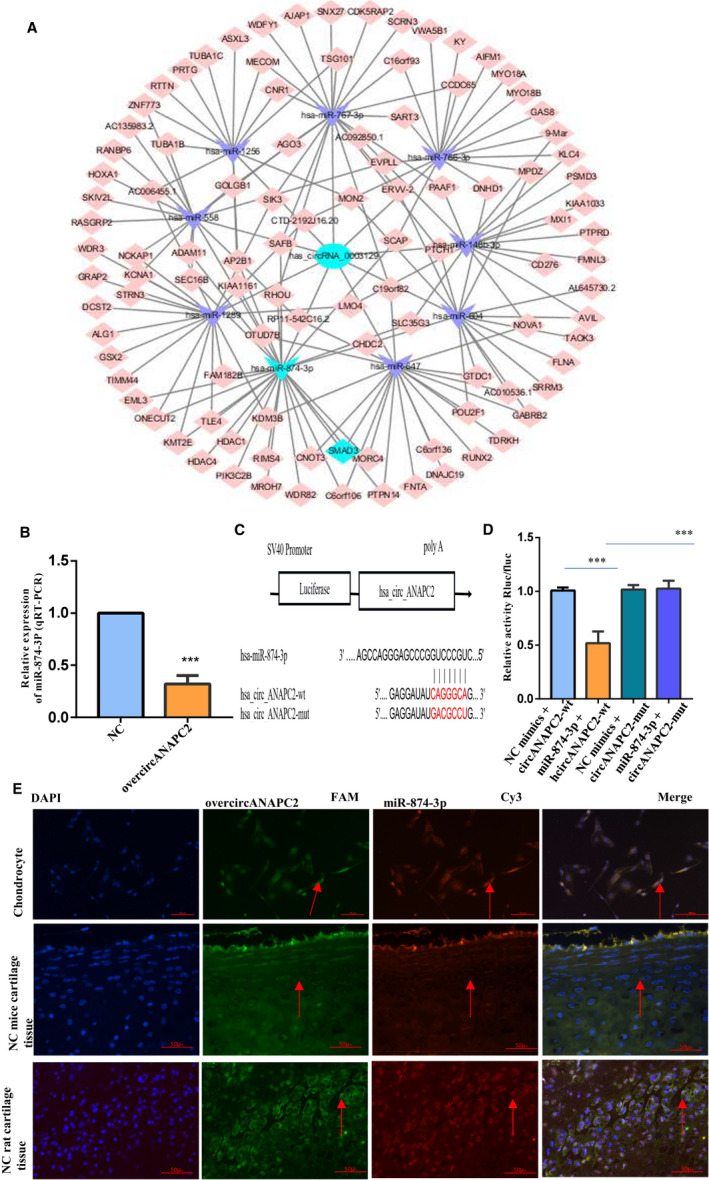
CircANAPC2 negatively regulates miR‐874‐3p. A, CircANAPC2 targeted miRNAs‐mRNAs network prediction. B, CircANAPC2 overexpression in human chondrocytes inhibits miR‐874‐3p expression. C, The potential target binding sites between circANAPC2 and miR‐874‐3p. D, Luciferase activity was repressed in human chondrocyte cotransfected with WT circANAPC2 and miR‐874‐3p mimics, while it was restored in cells cotransfected with Mut circANAPC2 and miR‐874‐3p mimics. E, In situ hybridization confirmed the co‐localization of circANAPC2 and miR‐874‐3p in the human chondrocyte and neonatal femur growth plate of C57 mice and SD rat. The data are presented as the mean ± SD. n = 3. ^***^ *P* <.001 vs control. ANAPC2, anaphase promoting complex subunit 2; mRNA, messenger RNA; miRNAs, microRNAs; and WT, wild‐type

Because circRNAs can function as a miRNA sponge and affect the expression levels of target genes, we analysed the possible interaction between circANAPC2 and miRNAs via Circinteractome database (http://circinteractome.nia. nih.gov/). As a result, 13 target genes were identified (hsa‐miR‐874‐3P, hsa‐miR‐146b‐3P, hsa‐miR‐1256, hsa‐miR‐1286, hsa‐miR‐558, hsa‐miR‐604, hsa‐miR‐647, hsa‐miR‐766 and hsa‐miR‐767‐3P). CircANAPC2 targeted miRNAs‐mRNAs network prediction was shown in Figure 4A. Finally, only 2 miRNAs (hsa‐miR‐874‐3P and hsa‐miR‐647) were down‐regulated following the overexpression of circANAPC2 (Figure 4B and Figure S4). MiR‐874‐3P was considered as the target gene of circANAPC2 owing to the highest differential expression of 4.08‐fold (Figure 4B). Subsequently, we observed that luciferase reporter activities were suppressed in the chondrocytes with wild‐type circANAPC2/miR‐874‐3p mimics cotransfection. However, after cotransfection with their mutation fragments, the luciferase activities were restored back (Figure 4 C and D), indicating that miR‐874‐3p is a possible target of circANAPC2 in ISS patients. Furthermore, in situ hybridization results verified the co‐localization of circANAPC2 and miR‐874‐3p in human chondrocytes as well as the dominant co‐localization in the proliferative area of mouse femoral growth plates and SD rat (Figure 4E).

**FIGURE 5:**
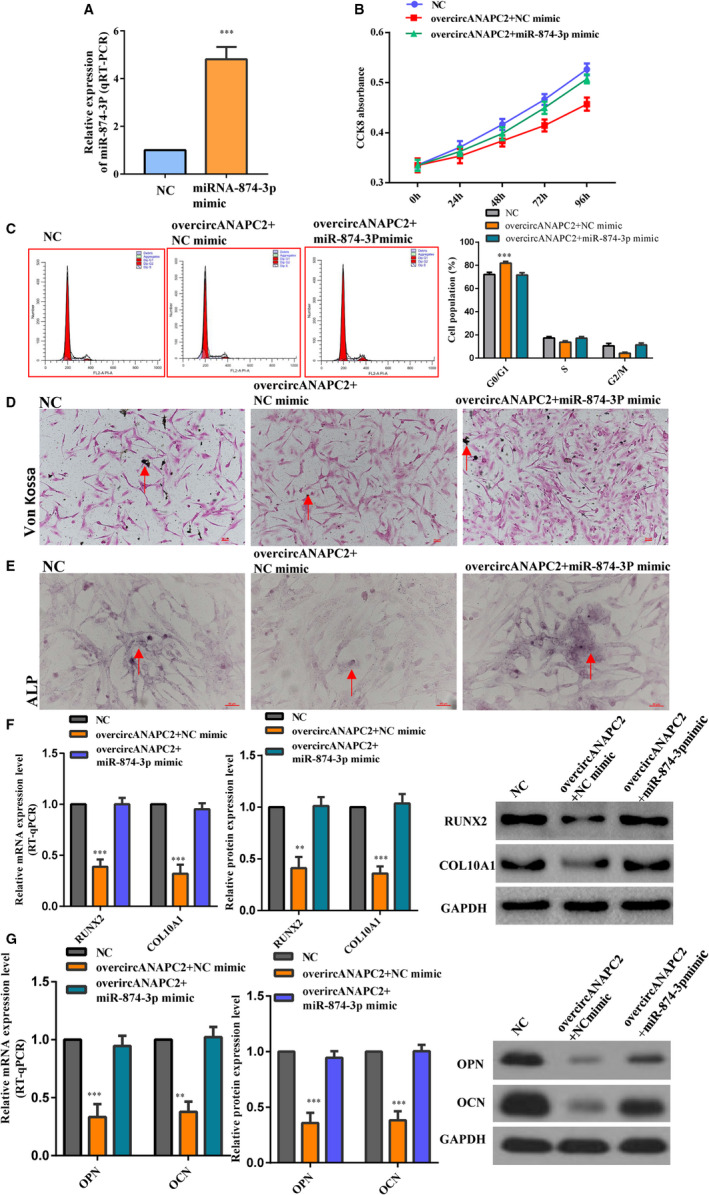
CircANAPC2 suppressed chondrocyte proliferation, hypertrophy and endochondral ossification by regulating miR‐874‐3p. A, MiR‐874‐3p mimics up‐regulated the expression of miR‐874‐3p. B,C, Although the chondrocyte proliferation was suppressed in the overcircANAPC2 + NC mimic group, it was reversed in the overcircANAPC2 + miR‐874‐3p mimic group. D, Von Kossa staining demonstrated reduced mineralization in overcircANAPC2 + NC mimic group. However, Von Kossa staining was recovered in the overcircANAPC2 + miR‐874‐3p mimic group. E, Like the Von Kossa staining, ALP activity was decreased in overcircANAPC2 + NC mimic group, but it also was recovered in the overcircANAPC2 + miR‐874‐3p mimic group. F, Although the mRNA and protein expressions of collagen type X and RUNX2 were down‐regulated in overcircANAPC2 + NC mimic group, no significant difference was observed in the overcircANAPC2 + miR‐874‐3p mimic group compared to the NC group. G, The mRNA and protein expressions of OPN and OCN were down‐regulated in overcircANAPC2 NC mimic group. However, no significant difference was observed in the overcircANAPC2 + miR‐874‐3p mimic group compared to the NC group. The data are presented as the mean ± SD. n = 3. Two groups were compared using *t* test or three groups were compared using ANOVA followed by Tukey's test. ^**^ *P* <.01, ^***^ *P* <.001 vs control. ANAPC2, anaphase promoting complex subunit 2; miR, microRNA; NC, negative control; ALP, alkaline phosphatase; OPN, osteopontin; OCN, osteocalcin; and RUNX2, runt‐related transcription factor 2

For further confirming the negative regulation between circANAPC2 and miR‐874‐3p, the expression levels of miR‐874‐3p were induced by microRNA mimics three days after circANAPC2 overexpression. The transfection efficiency of miR‐874‐3p mimics was assessed by qRT‐PCR (Figure 5A). Rescue experiment revealed that the high expression of miR‐874‐3p antagonized the suppression of endochondral ossification, hypertrophy and chondrocyte growth caused by circANAPC2 overexpression (Figure 5B‐5G). For example, although the chondrocyte proliferation was suppressed in overcircANAPC2 + NC mimic group, it was reversed in overcircANAPC2 + miR‐874‐3p mimic group (Figure 5B and C). Von Kossa staining revealed that mineralization was reduced in overcircANAPC2 + NC mimic group. However, Von Kossa staining was recovered in the overcircANAPC2 + miR‐874‐3p mimic group (Figure 5D). Similar to Von Kossa staining, ALP activity was decreased in overcircANAPC2 + NC mimic group, but it also was recovered in overcircANAPC2 + miR‐874‐3p mimic group (Figure 5E). Although the mRNA and protein expression levels of RUNX2 and collagen type X were down‐regulated in overcircANAPC2 + NC mimic group, no significant difference was observed in overcircANAPC2 + miR‐874‐3p mimic group compared to NC group (Figure 5F). The mRNA and protein expression levels of OPN and OCN were down‐regulated in overcircANAPC2 NC mimic group. However, no significant difference was observed in overcircANAPC2 + miR‐874‐3p mimic group compared to NC group (Figure 5G). Taken together, these results indicated that circANAPC2 suppressed endochondral ossification, hypertrophy and chondrocyte proliferation via regulation of miR‐874‐3p.

### 3.5 CircANAPC2 significantly increases SMAD3 expression, but not SMAD2 expression, by inhibiting miR‐874‐3p

**FIGURE 6:**
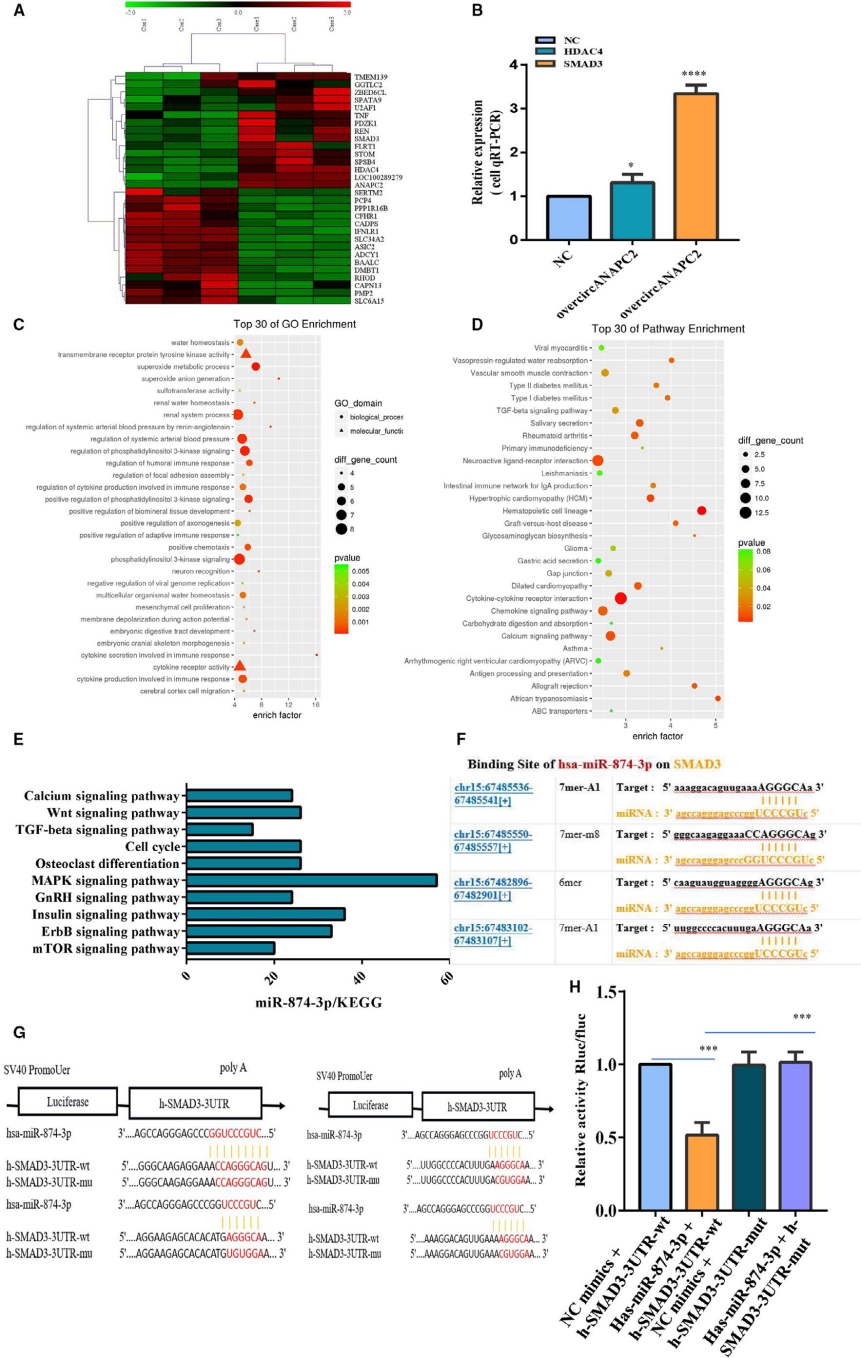
mRNA high‐throughput sequencing and bioinformatics analyses following overcircANAPC2, and luciferase activity between miR‐874‐3p and SMAD3. A, The top 30 differentially expressed mRNA was shown. B, SMAD3 demonstrated the highest different expressions and the excellent reliability was observed between mRNA microarray and RT‐qPCR following overcircANAPC2. C,D, The top 30 KEGG pathway and GO enrichment are exhibited. The TGF‐β signal pathways is closely associated with chondrocyte proliferation, hypertrophy and endochondral ossification. SMAD3 is a key gene of the TGF‐β signal pathways. E, The miR‐874‐3p target genes were predicted using Starbase database, and KEGG pathway analyses were performed on these genes; 136 targeted signalling pathways were identified (*P* <.05). Ten of 136 signal pathways were associated with chondrocyte development and chondral ossification. SMAD3 participates in three of these pathways (TGF‐β, Wnt and cell cycle). F, Starbase database shows four binding sites with high scores between SMAD3 and miR‐874‐3p. G,H, Luciferase activity was repressed in human chondrocyte cotransfected with WT SMAD3 and miR‐874‐3p mimics, while it was restored in cells cotransfected with Mut SMAD3 and miR‐874‐3p mimics. The data are presented as the mean ± SD. n = 3. ^***^ *P* <.001 vs control. ANAPC2, anaphase promoting complex subunit 2; mRNA, messenger RNA; SMAD3, SMAD family member 3; TGF‐β, transforming growth factor‐β*;* RT‐qPCR, reverse transcription‐quantitative PCR; KEGG, Kyoto Encyclopedia of Genes and Genomes GO, Gene ontology; Wnt, wingless/integrated; Mut, mutant; and WT, wild‐type

To identify the target genes of miR‐874‐3p, the differentially expressed mRNA was analysed via mRNA high‐throughput sequencing after overexpression of circANAPC2 (Figure 6A). The top five mRNA of up‐regulation with the highest different expressions were verified by RT‐qPCR. Finally, SMAD3 and HDAC4 demonstrated the excellent reliability between mRNA microarray and RT‐qPCR (Figure 6B). However, SMAD3 present the most significant difference (3.65‐fold) following overcircANAPC2 (Figure 6B). The top 30 KEGG pathway and GO enrichment are exhibited on the basis of the differentially expressed mRNA (Figure 6C and D). The TGF‐β signal pathways are closely associated with chondrocyte proliferation, hypertrophy and endochondral ossification. SMAD3 is a key gene of the TGF‐β signal pathways. Subsequently, we predicted the target mRNA of miR‐874‐3p using Starbase database, and their corresponding signalling pathways were analysed by KEGG pathway enrichment. In total, we identified 136 targeted signalling pathways according to *P*‐values of less than 0.05 (Supplementary Excel). Ten of 136 signal pathways were associated with chondrocyte development and chondral ossification (Figure 6E). Interestingly, SMAD3 is involved in three of these pathways (cell cycle, TGF‐β and Wnt). Moreover, SMAD3 and miR‐874‐3p were estimated to have four binding regions with higher scores (Figure 6F). Thus, SMAD3 was selected as the key gene for ISS pathogenesis.

**FIGURE 7:**
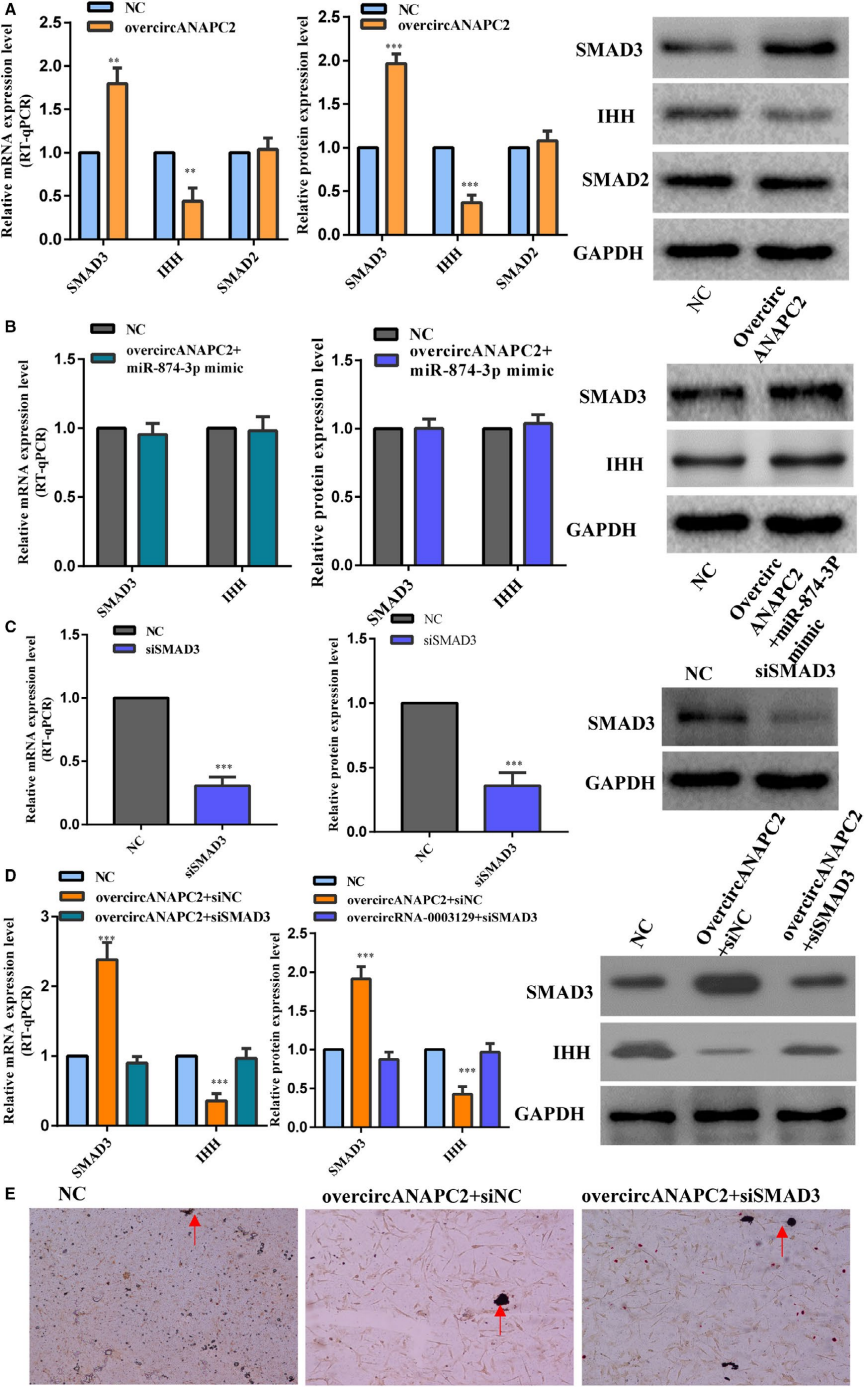
CircANAPC2 suppressed chondrocyte proliferation, hypertrophy and endochondral ossification by regulating miR‐874‐3p/ SMAD3, not SMAD2. A, SMAD3 exhibited up‐regulation compared to the control following overcircANAPC2. Meanwhile, the expression of IHH, a SMAD3 target gene, was down‐regulated compared to the control. No significant difference was observed in the SMAD2 between the overcircANAPC2 group and normal control group. B, The up‐regulated SMAD3 and the down‐regulated IHH were reversed in the overcircANAPC2 + miR‐874‐3p mimic group, and no significant difference was observed in the SMAD3 and IHH between the overcircANAPC2 + miR‐874‐3p mimic group and normal control group. This rescue experiment further confirmed the miR‐874‐3p negatively regulates SMAD3. C, The effectiveness of SMAD3 siRNA was detected in the human chondrocyte by RT‐qPCR and Western blot. D, Although SMAD3 presented up‐regulation and IHH showed down‐regulation in the overcircANAPC2 + siNC, it did not demonstrate significant difference between the overcircANAPC2 + siSMAD3 and normal control group. E, Von Kossa staining demonstrated reduced mineralization in the overcircANAPC2 + siNC group. However, Von Kossa staining was recovered in the overcircANAPC2 + siSMAD3 group. This indicated that circANAPC2 suppressed chondrocyte proliferation, hypertrophy and endochondral ossification by regulating miR‐874‐3p/ SMAD3 axis. The data are presented as the mean ± SD. n = 3. Two groups were compared using t test or three groups were compared using ANOVA followed by Tukey's test. ^**^ *P* <.01, ^***^ *P* <.001 vs control. ANAPC2, anaphase promoting complex subunit 2; mRNA, messenger RNA; SMAD2, SMAD family member 2; SMAD3, SMAD family member 3; TGF‐β, transforming growth factor‐β*;* RT‐qPCR, reverse transcription‐quantitative PCR; siNC, negative control siRNA; siSMAD3, SMAD family member 3 siRNA; miR, microRNA; and IHH, Indian hedgehog homolog

Luciferase reporter assay showed the reduction in HEK293 cells cotransfected with SMAD3‐WT/miR‐874‐3p mimics, and it was restored after the SMAD3‐Mut and miR‐874‐3p mimics were cotransfected (Figure 6G and H). As expected, SMAD3 exhibited up‐regulation and the expression of IHH, a SMAD3 target gene, was down‐regulated in the overcircANAPC2 group (Figure 7A). There were no remarkable differences in the expression levels of SMAD2 and IHH between the overcircANAPC2 + miR‐874‐3p group and normal control group (Figure 7A). The rescue experiment of miR‐874‐3p indicated that the up‐regulated SMAD3 and the down‐regulated IHH following overcircANAPC2 were reversed in the overcircANAPC2 + miR‐874‐3p mimic group (Figure 7B). This further confirmed the circANAPC2 significantly increases SMAD3 expression, but not SMAD2, by suppressing miR‐874‐3p.

### 3.6 CircANAPC2 suppressed endochondral ossification, hypertrophy and chondrocyte proliferation by regulating miR‐874‐3p/SMAD3

RT‐qPCR and Western blot verified the effectiveness of SMAD3 siRNA in the human chondrocyte (Figure 7C). SMAD3 presented up‐regulation and IHH showed down‐regulation in the overcircANAPC2 compared to normal control group. Meanwhile, Von Kossa staining also demonstrated mineralization was attenuated in the overcircANAPC2 + siNC group (Figure 7E). However, it did not demonstrate significant difference in SMAD3 and IHH between the overcircANAPC2 + siSMAD3 and normal control group (Figure 7D). Moreover, Von Kossa staining was also recovered in the overcircANAPC2 + siSMAD3 group (Figure 7E). This indicated that circANAPC2 suppressed endochondral ossification, hypertrophy and chondrocyte proliferation by regulating miR‐874‐3p/ SMAD3 axis.

### 3.7 Down‐regulation of circANAPC2 enhanced chondrocyte hypertrophy and endochondral ossification by regulating miR‐874‐3p/SMAD3

**FIGURE 8:**
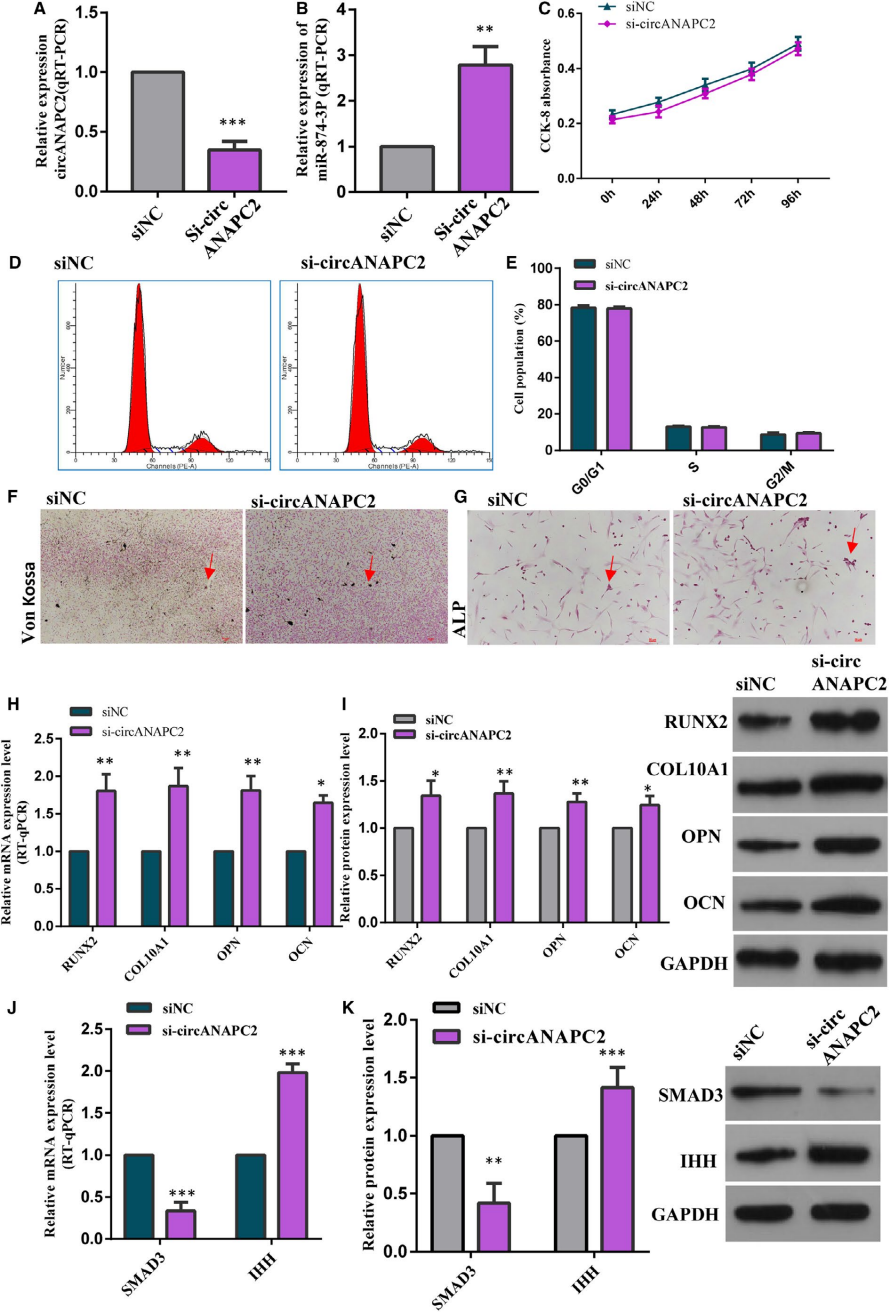
Down‐regulation of circANAPC2 enhanced chondrocyte hypertrophy and endochondral ossification by regulating miR‐874‐3p/SMAD3. A and B, The expression of miR‐874‐3p was up‐regulated after circANAPC2 down‐regulation by siRNA. C‐E, No significant difference was observed in the proliferation of human chondrocytes as measured by CCK‐8 and flow cytometry assays between the group of circANAPC2 down‐regulation and control group. F and G, ALP activity was increased and Von Kossa staining demonstrated increased mineralization. H and I, The expressions of collagen type X, RUNX2, OCN and OPN were significantly up‐regulated following circANAPC2 down‐regulation. J and K, SMAD3 was down‐regulated and IHH was up‐regulated following circANAPC2 down‐regulation. The data are presented as the mean ± SD. n = 3. Two groups were compared using t test or three groups were compared using ANOVA followed by Tukey's test. ^**^ *P* <.01, ^***^ *P* <.001 vs control. ANAPC2, anaphase promoting complex subunit 2; siCircANAPC2, circANAPC2 siRNA; siNC, negative control siRNA; miR, microRNA; SMAD3, SMAD family member 3; IHH, Indian hedgehog homolog; ALP, alkaline phosphatase; OPN, osteopontin; OCN, osteocalcin; and RUNX2, runt‐related transcription factor 2

The expression of miR‐874‐3p was up‐regulated after circANAPC2 down‐regulation by siRNA (Figure 8A and B). Meanwhile, SMAD3 was down‐regulated and IHH was up‐regulated following circANAPC2 down‐regulation (Figure 8J and K). Though no obvious difference was found in the proliferation of human chondrocytes as determined by CCK‐8 and flow cytometric assays between the circANAPC2 down‐regulation group and control group (Figure 8C‐E), the expressions of collagen type X, RUNX2, OCN and OPN were significantly up‐regulated following circANAPC2 down‐regulation (Figure 8H and I). Moreover, ALP activity was also increased (Figure 8G **)** and Von Kossa staining demonstrated increased mineralization (Figure 8F **)**. These results indicated that circANAPC2 down‐regulation can markedly enhance chondrocyte hypertrophy and endochondral ossification.

## 4 DISCUSSION

CircRNAs have been increasingly recognized as diagnostic and therapeutic biomarkers for many diseases. This is primarily owing to their high stability than other non‐coding RNA, such as miRNAs and lncRNAs. Although identifying the differentially expressed circRNAs on the growth plate of ISS patients compared to that of normal children is the most superior strategy for uncovering the molecular mechanisms of the disease, the samples of growth plate of ISS children cannot be obtained owing to principles of medical ethics. Numerous studies have shown that circRNAs are expressed in human blood.

Meanwhile, the aberrant circRNA expression of peripheral blood samples has also been closely related with many diseases. Zhou et al^27^ observed that circRNA ciRS‐7 was down‐regulated and miR‐7 was up‐regulated in the peripheral blood samples of osteoarthritis patients. Subsequent studies indicate that the ciRS‐7 can promote apoptosis and inflammation of chondrocytes in osteoarthritis by acting as sponges with miR‐7. This may provide a novel therapeutic target for osteoarthritis. Meanwhile, Wang et al^28^ found 1,627 differentially expressed circRNAs in osteoarthritis vs Kashin‐Beck disease patients. Down‐regulated circRNAs_0020014 was verified by qRT‐PCR. The ROC curve demonstrated that circ_0020014 of peripheral blood could be considered as a promising biomarker for differential diagnosis between osteoarthritis vs Kashin‐Beck disease. Haque et al^29^ and Wang et al^30^ also found that circRNAs were stably presented in peripheral venous blood and associated with age‐related disease and hepatocellular carcinoma. However, there is a lack of research regarding the possible role of aberrant circRNAs expression in mediating ISS pathogenesis.

In this study, the authors first confirmed that circANAPC2 was up‐regulated in ISS patients compared to normal children. Meanwhile, the expression of circANAPC2 also demonstrated the reliability between microarrays and RT‐qPCR. Subsequent investigation showed that overexpression of circANAPC2 suppressed chondrocyte proliferation, hypertrophy and endochondral ossification. The bioinformatics analysis revealed that circANAPC2 could be targeted by miR‐874‐3p; and circANAPC2 overexpression in chondrocytes inhibits the expression of miR‐874‐3p. The results of luciferase reporter assay further confirmed that miR‐874‐3p is a key target of circANAPC2.

Lin et al^31^ confirmed the expression of miR‐874 in rat osteoblasts and found that miR‐874 can target SUFU, thereby promoting the proliferation and differentiation of osteoblasts in osteoporosis rats. However, the expression and functions of miR‐874‐3p in cartilage and growth plate were not addressed. Therefore, we detected the expression levels of circANAPC2 and miR‐874‐3p in the chondrocytes and growth plate. The authors first demonstrated the co‐localization of circANAPC2 and miR‐874‐3p in the human chondrocytes and murine femoral growth plates via in situ hybridization. Rescue experiment revealed that the high expression of miR‐874‐3p antagonized the suppression of endochondral ossification, hypertrophy and chondrocyte growth caused by circANAPC2 overexpression. This suggests that circANAPC2 suppresses endochondral ossification, hypertrophy and chondrocyte proliferation via miR‐874‐3p.

For further identifying the target genes of miR‐874‐3p, we performed a high‐throughput screening of mRNA expression after overexpression of circANAPC2 in the human chondrocytes. RT‐qPCR and luciferase reporter assays showed that miR‐874‐3p could be targeted by Smad3, and miR‐874‐3p can down‐regulate the expression of Smad3. Li et al^32^ reported that the expression of collagen type X and ALP activity in chondrocytes can be suppressed following overexpression of Smad3. Wang et al^33^ found that Smad3 can block the suppression of IHH promoter activity by TGFβ. As a result, Smad3 inhibited IHH expression in the newborn growth plates. Jia et al^22^ also demonstrated that Smad3 can inhibit endochondral ossification of growth plate by down‐regulating IHH expression in the DDH rabbit. Several studies^34^, ^35^, ^36^, ^37^ have indicated that IHH down‐regulation not only can inhibit the chondrocyte growth and differentiation, but also can impair osteoblastogenesis during endochondral ossification. To reveal the role of Smad3 on the circANAPC2/ miR‐874‐3p axis in our study, rescue experiments of Smad3 were performed. As expected, we observed that up‐regulating the expression of Smad3 inhibited the expression of collagen type X, reduced ALP activity and suppressed the expression of IHH. Meanwhile, the suppression of endochondral ossification, hypertrophy and chondrocyte proliferation after circANAPC2 overexpression was reversed by the up‐regulation of Smad3. The rescue experiment results indicated that circANAPC2 suppresses endochondral ossification, hypertrophy and chondrocyte proliferation via the miR‐874‐3p/Smad3 axis.

Besides Smad3, the authors found that HDAC4 was also up‐regulated after overcircANAPC2 in the present study. Kang et al^38^ and Liu et al^39^ reported that both HDAC4 and Smad3 can repress the expression of Runx2, a marker gene of osteoblast differentiation, and inhibit its function. Subsequently, Yu et al^40^ observed that pSmad3 can recruit HDAC4 in the nucleus. As a result, Runx2 expression was inhibited by the complexes. Though Smad3 presented the most significant difference (3.65‐fold) in our study and the rescue experiment of Smad3 supported that circANAPC2 suppresses endochondral ossification, hypertrophy and chondrocyte proliferation via the miR‐874‐3p/Smad3 axis, it is still possible that HDAC4 as a cooperative factor participate in the inhibition of endochondral ossification by down‐regulating Runx2 expression.

There still is limitation in our study. The authors observed that the expression of circANAPC2 was up‐regulated in ISS patients and found that circANAPC2 can inhibit chondrocyte proliferation, hypertrophy and endochondral ossification via a novel axis circANAPC2/ miR‐874‐3p/SMAD3 in vitro. However, these results need to be verified in vivo via knockout or overexpression animal models. In conclusion, our study reveals that circANAPC2 can bind to miR‐874‐3p and regulate SMAD3 signalling pathway, thereby contributing to the pathogenesis of ISS.

## CONFLICT OF INTEREST

The authors have no conflicts of interest to declare.

## AUTHOR CONTRIBUTIONS

**Xijuan Liu:** Conceptualization (equal); Data curation (equal); Formal analysis (equal); Funding acquisition (equal); Methodology (equal); Software (equal); Validation (equal); Writing–original draft (equal); Writing–review & editing (equal). **Zhi Du:** Data curation (equal); Investigation (equal); Methodology (equal); Software (equal); Validation (equal); Writing–review & editing (equal). **Xuan Yi:** Data curation (equal); Formal analysis (equal); Investigation (equal); Methodology (equal); Resources (equal); Software (equal). **Tianle Sheng:** Data curation (equal); Formal analysis (equal); Investigation (equal); Methodology (equal); Software (equal); Writing–original draft (equal). **Jinghong Yuan:** Data curation (equal); Formal analysis (equal); Investigation (equal); Methodology (equal); Resources (equal); Validation (equal); Visualization (equal). **Jingyu Jia:** Conceptualization (equal); Data curation (equal); Formal analysis (equal); Funding acquisition (equal); Investigation (equal); Project administration (equal); Resources (equal); Software (equal); Supervision (equal); Validation (equal); Visualization (equal); Writing–original draft (equal); Writing‐review & editing (equal).

## Supporting information

Fig S1

Fig S2

Fig S3

Fig S4

Table S1

## Data Availability

Data in this research for supporting the results are all included within the article. The results of microarray analysis have been deposited to GEO (GEO accession number: GSE157147).
