# Supplementary figures and images for: Circular RNA circANAPC2 mediates the impairment of endochondral ossification by miR‐874‐3p/SMAD3 signalling pathway in idiopathic short stature
Source: J Cell Mol Med. 2021 Mar 13;25(7):3408–26. doi: 10.1111/jcmm.16419 (PMC8034469; doi:10.1111/jcmm.16419)

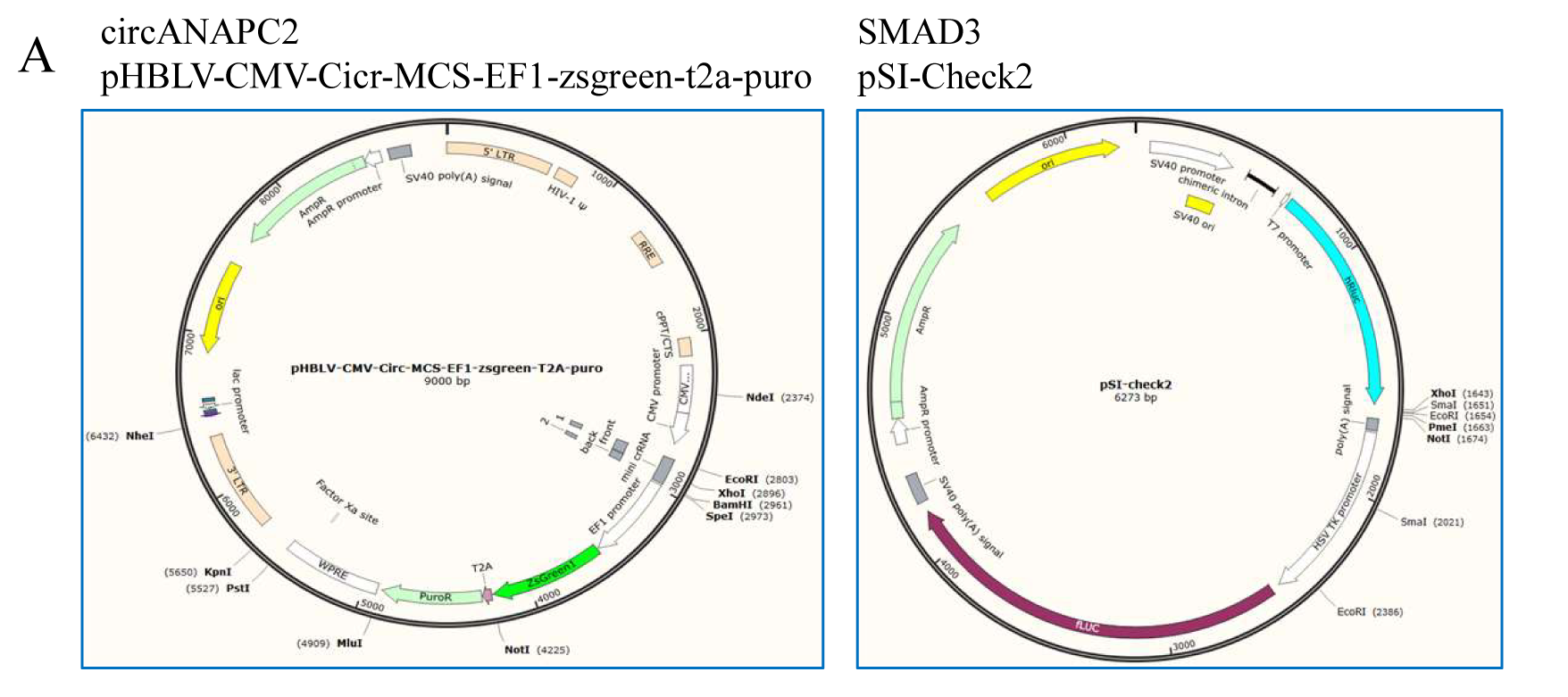

Supplement: Supplementary file 1 — Fig S1 [file JCMM-25-3408-s003.png]

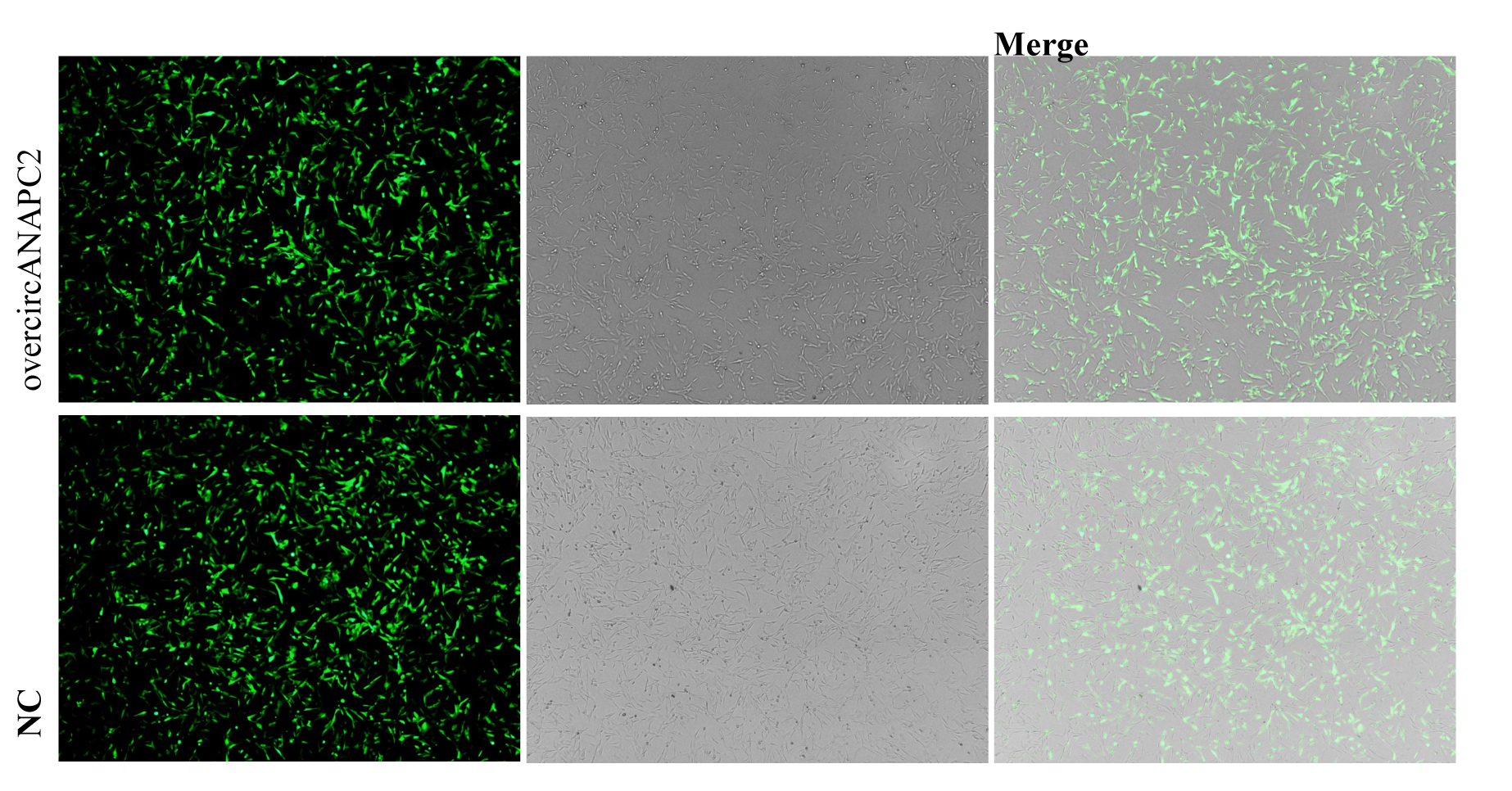

Supplement: Supplementary file 2 — Fig S2 [file JCMM-25-3408-s004.png]

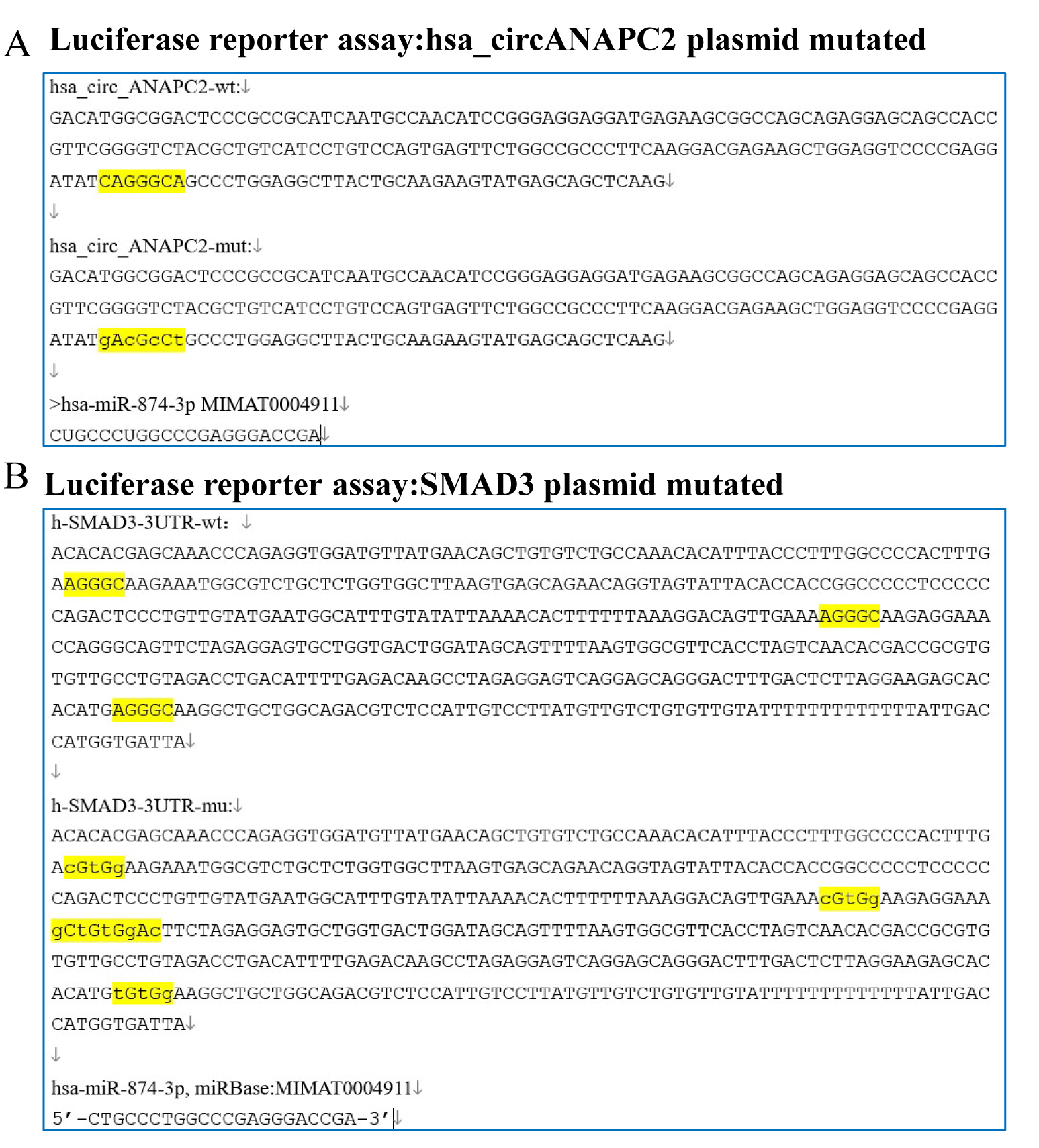

Supplement: Supplementary file 3 — Fig S3 [file JCMM-25-3408-s002.png]

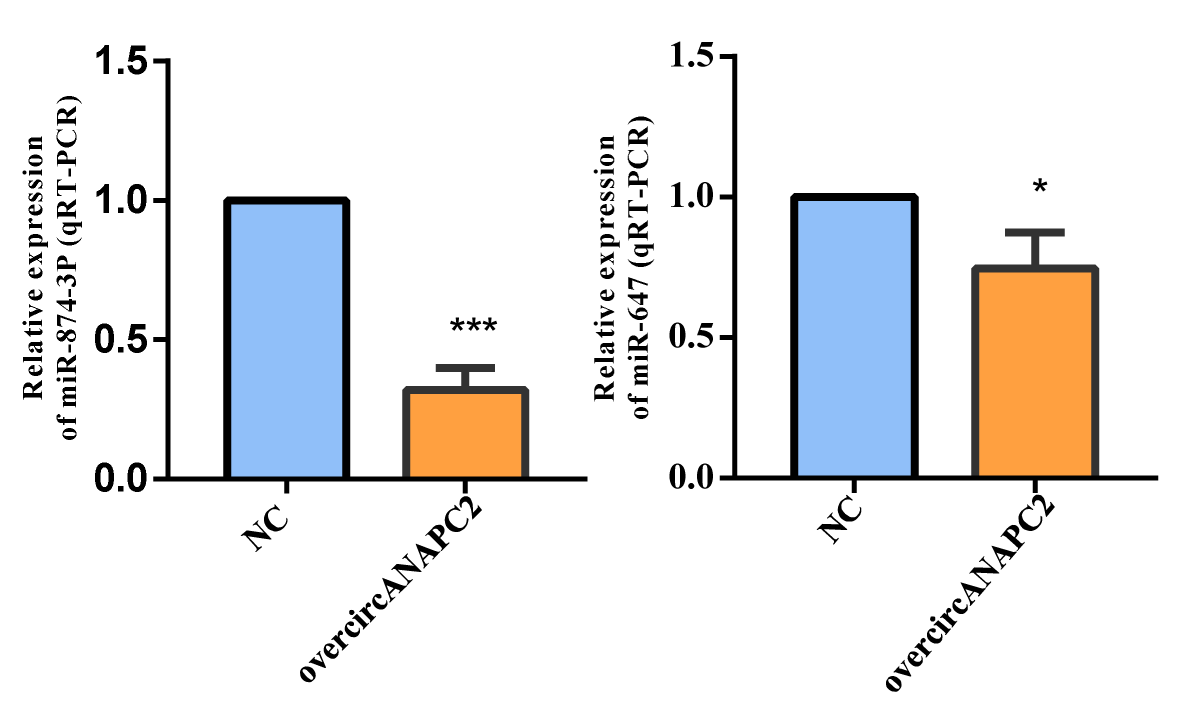

Supplement: Supplementary file 4 — Fig S4 [file JCMM-25-3408-s001.png]
